# Analysis of Forward Trunk Bending in Women with Chronic Low Back Pain Undergoing Functional Training

**DOI:** 10.3390/jcm14124156

**Published:** 2025-06-11

**Authors:** Aleksandra Adamik, Piotr Krężałek, Edyta Mikołajczyk

**Affiliations:** 1 Institute of Applied Sciences, Faculty of Motor Rehabilitation, University of Physical Culture, 31-571 Krakow, Poland; 2 Laboratory of Biophysics and Movement Analysis, Department of Biomechanics, University of Physical Culture, 31-571 Krakow, Poland

**Keywords:** functional training, motion analysis, chronic pain

## Abstract

**Background/Objectives:** This paper analyzes the movement and relationships within the lumbopelvic–hip complex during forward trunk bending in young women with chronic low back pain. **Methods**: This study involved 24 women aged 20–24 with chronic low back pain. They were randomly divided into two equal-sized groups: Group 1 participated in a 12-week functional training program, and Group 2 was a control without any intervention. The level of perceived pain was assessed using the Visual Analog Scale (VAS). Qualitative motion analysis was performed using the BTS SMART-D system. Custom indicators were developed to evaluate the angular relationships and ranges of motion in the lumbar spine and the lumbopelvic–hip complex. The functional training program consisted of three sections: motor control and stabilization, muscle strengthening, and stretching exercises. Statistical analysis was performed using Statistica 13.3. **Results**: The therapy resulted in a reduction in perceived pain levels reported by the participants. There was a significant improvement in the quality of the forward trunk bending pattern in women who underwent functional training. **Conclusions**: Significant modifications in the quality, technique, and angular relationships within the lumbopelvic–hip complex were observed during the forward trunk bending pattern in women undergoing functional training. It has proven to be an effective form of therapy for chronic low back pain.

## 1. Introduction

Pain symptoms lasting at least three months, classified as chronic pain, affect 70–85% of the population at least once in their lifetime, with a predominance of incidence in women (61.4%). This indicates the large scale and complexity of the problem. It should be noted that chronic pain affects not only the physical sphere and functional capacity of individuals, but also encompasses pathoanatomical and psychological factors [1]. Nowadays, it is necessary to pay attention to factors related to work ergonomics, the quality of performed movements, and the overall well-being of patients.

Reduced physical activity in individuals with chronic pain can lead to higher-level adaptations, such as cortical reorganization in the central nervous system. Along with the fear of pain during an activity that previously triggered it, attention is redirected toward more conscious strategies of central movement preparation [2,3,4]. Patients’ electroencephalography (EEG) measurements show reduced cortical inhibition in pain control regions [5]. The primary somatosensory cortex, which encodes the sensory perception of pain, its location, and duration, is excessively stimulated in chronic pain cases. In response to stimuli from peripheral neurons exhibiting activity-dependent plasticity (long-term excitation or inhibition), constant reorganization occurs in the cortex, and slower EEG frequencies may play a key role in the abnormal processing of pain [6,7,8].

The vast majority of patients with chronic low back pain do not require surgical intervention, and the increasing access to physiotherapy and screening tools allowing for predicting the dynamics of pain development may have a positive impact on the choice of treatment strategies [9]. The development of technology allows for the use of movement optimization and analysis methods. One of them is the comprehensive motion analysis system, including BTS SMART-D, used in this research, which enables recording body movements and assessing kinesthetic parameters in healthy individuals, athletes, and patients [10].

Given the complexity of the problem of chronic low back pain, qualitative motion analysis, which can directly influence the acceleration of degenerative changes in the musculoskeletal system, seems justified for the effective and lasting treatment and prevention of back pain development. Our research is the first to present the use of a diagnostic tool for comprehensive biomechanical motion analysis, enabling the subsequent planning of training as a form of rehabilitation and the later evaluation of its effects, clearly indicating the beneficial changes that occurred in the execution of the functional pattern in the study participants.

This study analyzed the movement and relationships within the lumbopelvic–hip complex during forward trunk bending in young women with chronic low back pain who participated in a functional training program.

## 2. Materials and Methods

### 2.1. Study Design and Participants

The study was conducted from January to May 2023; it involved 24 women aged 20–24 ($\bar{x}$ = 21.92). The participants were randomly assigned to two equal-sized groups. Group 1 (G1) participated in 12-week therapy consisting of functional training of the lumbopelvic–hip complex, while Group 2 (G2) was the control without any intervention. All participants were assessed twice during the study, i.e., before and after the 12 weeks, at the Biophysics and Movement Analysis Laboratory. None of the participants engaged in regular physical activity. The inclusion criteria required chronic (lasting more than three months) low back pain rated ≥ 3/10 on the Visual Analog Scale (VAS), normal BMI, good overall health, and no history of abdominal, spinal, or lower limb injuries, fractures, or surgeries. The level of low back pain was estimated using a Visual Analog Scale (VAS) ranging from 0 to 10, where 0 indicates no pain, and 10 indicates the highest possible pain intensity [11].

Details regarding the basic characteristics of the women enrolled in the study are presented in Table 1.

### 2.2. Ethics Committee Approval

The study was approved by the Ethics Committee of the Regional Medical Chamber in Krakow, No. 158/KBL/OIL/2022.

### 2.3. Functional Training of the Lumbopelvic–Hip Complex

The functional training of the lumbopelvic–hip complex was scheduled for 12 consecutive weeks, with sessions held three times per week. The training plan included three separate sections focusing on motor control and stabilization, muscle strengthening, and stretching exercises. Exercises were supervised by a physiotherapist once a week, and participants conducted sessions independently at home twice a week. The program significantly emphasized teaching participants motor control skills, including neutral pelvic positioning and correct execution of functional patterns (Supplementary Materials).

### 2.4. Measurements

**Figure 1:**
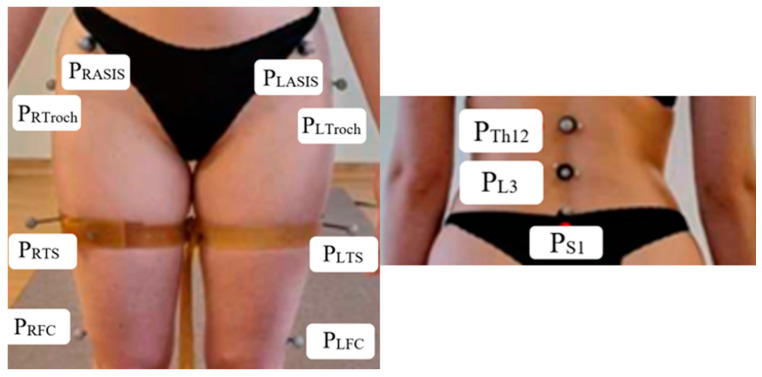
Placement of markers: front and rear view.

The BTS SMART-D system (BTS Bioengineering, Milan, Italy), equipped with six cameras operating at a sampling frequency of 70 Hz, enabled three-dimensional motion analysis. Angular relationships and ranges of motion of the lumbar spine and lumbopelvic–hip complex were evaluated. The positions of individual markers during motion were recorded using the BTS SMART Capture module. The study was conducted with participants standing in undergarments, allowing markers to be placed on selected anatomical points. Participants performed forward trunk bending movements. The final analysis used the average of three measurements. After recording, the markers were identified using the BTS SMART Tracker module. Smoothed marker coordinate data were used to calculate angles in the BTS SMART Analyzer module. Based on the assumptions of other authors [12,13], 11 markers were placed according to the study scheme, including the following: P_S1_—S1 spinous process, P_L3_—L3 spinous process, P_Th12_—Th12 spinous process, P_RASIS_—right anterior superior iliac spine, P_LASIS_—left anterior superior iliac spine, P_RTroch_—right greater trochanter of the femur, P_LTroch_—left greater trochanter of the femur, P_RTS_—right thigh stick marker, P_LTS_—left thigh stick marker, P_RFC_—right lateral femoral condyle, P_LFC_—left lateral femoral condyle. The placement of markers from the front and rear view is presented in Figure 1.

**Figure 2:**
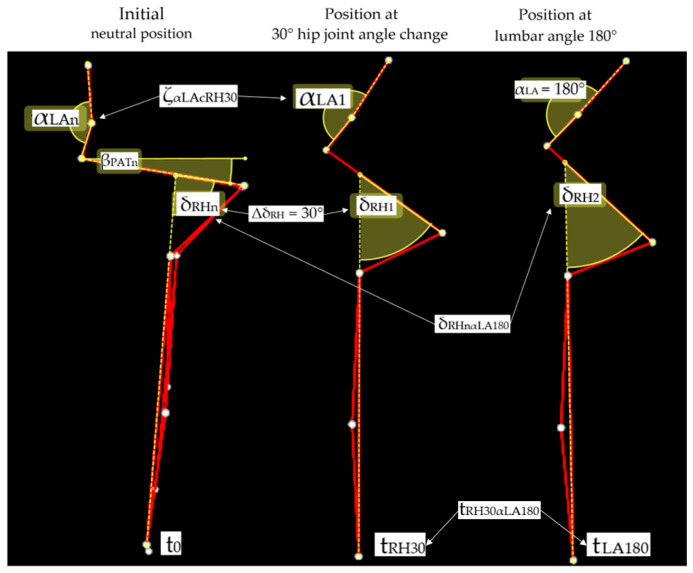
Visual representation of the three key positions analyzed during forward trunk bending movement using the BTS SMART-D system. The initial neutral position (**left**) shows the starting posture, indicating lumbar angle (αLAn) and pelvic anterior tilt angle (βPATn). The (**middle**) image represents the position when the right hip joint angle changes by 30° from the neutral position (ΔδRH = 30°), showing the corresponding change in the lumbar angle (ζαLAcRH30). The (**right**) image depicts the position at which the lumbar angle reaches 180° (αLA = 180°). The time variables (t0, tRH30, tLA180) indicate the temporal measurement points, while δRHnαLA180 represents the total change in the right hip joint angle between the neutral position and the position reached at a lumbar angle of 180°. White dots represent reflective markers placed at anatomical landmarks, connected by red lines showing the body segments used for angle calculations. Both δRH1 and δRH2 shown in the figure represent the hip joint angles used to calculate relative angle changes from the neutral position.

In the analyzed sagittal plane, angles formed by individual segments of the model were computed. Figure 2 illustrates the key measurement positions and angular relationships analyzed during the forward trunk bending. Three positions are of particular interest: the initial neutral position, the position at 30° hip joint angle change, and the position at lumbar angle 180°. The pelvic ring segment was defined using the positions of points: P_RASIS_, P_LASIS_, P_S1_ (including the midpoint of the line connecting P_RASIS_ and P_LASIS_-P_MidASIS_). The lumbar angle (α_LAn_) was determined based on markers P_S1_, P_L3_, and P_Th12_. The anterior pelvic tilt angle (β_PATn_) was calculated using P_S1_ and P_MidASIS_. Markers on the thigh and pelvis were used to determine the orientation of these segments and track angular changes in the hip joints during movement in relation to the lumbar angle (α_LAn_), thereby recording qualitative variables in the entire lumbopelvic–hip complex during forward trunk bending.

The variables t_LH30αRA180_ and t_LH30αLA180_ indicate the time difference between the moment the right and left hip joint angle reached 30° flexion and the moment the lumbar angle reached 180°. The variables ζ_αLAcRH30_ and ζ_αLAcLH30_ indicate a change in lumbar angle relative to the neutral position when the hip joint flexed by 30° relative to the initial angle. The variables δ_RHnαLA180_ and δ_LHnαLA180_ represented the change in the right and left hip joint angles between the neutral position and position reached at a lumbar angle of 180° (Figure 2).

As shown in Figure 2, these variables enable a comprehensive analysis of temporal and angular relationships within the lumbopelvic–hip complex during forward trunk bending, focusing on the coordination between lumbar spine movement and hip joint flexion.

### 2.5. Statistical Analysis

The study was designed in compliance with the CONSORT (Consolidated Standards of Reporting Trials) guidelines to ensure transparency and reproducibility of clinical trial reporting. Due to the exploratory nature of the study and strict inclusion criteria (age, sex, health status), the sample size (*n* = 24) was determined based on the availability of eligible participants within the specified recruitment timeframe. Although no formal power analysis was conducted, effect size values (d) were calculated for key outcomes to estimate the magnitude of the intervention’s impact.

Statistical analyses were conducted using Statistica 13.3 software (StatSoft, Tulsa, OK, USA). Measurement results were processed using descriptive statistical methods, including arithmetic means ($\bar{x}$), medians (Me), minimums (Min), maximums (Max), and standard deviations (SD). Statistical significance was set at *p* < 0.05. Before comparative analyses, all variables were tested for normality using the Shapiro–Wilk test. Based on its results, both parametric (Student’s *t*-test for independent and dependent samples) and nonparametric tests (Mann–Whitney U and the Wilcoxon signed-rank test) were appropriately applied. The choice of test depended on the distribution characteristics of the specific variables. Effect size (d) was calculated using Cohen’s d formula. For between-group comparisons, d was computed as the standardized difference between two means divided by the pooled standard deviation. For within-group comparisons (pre–post measurements), d was calculated based on the mean differences and their standard deviations. This approach allows for assessment of the practical significance of the observed differences, which is particularly valuable given the small sample size and the exploratory nature of the study. Cohen classified effect sizes as small (d = 0.2), medium (d = 0.5), and large (d ≥ 0.8) [14]. Values exceeding 1.2 can be considered very large effects, while those above 2.0 represent extremely large effects.

### 2.6. Flow Diagram of Participants

**Figure 3:**
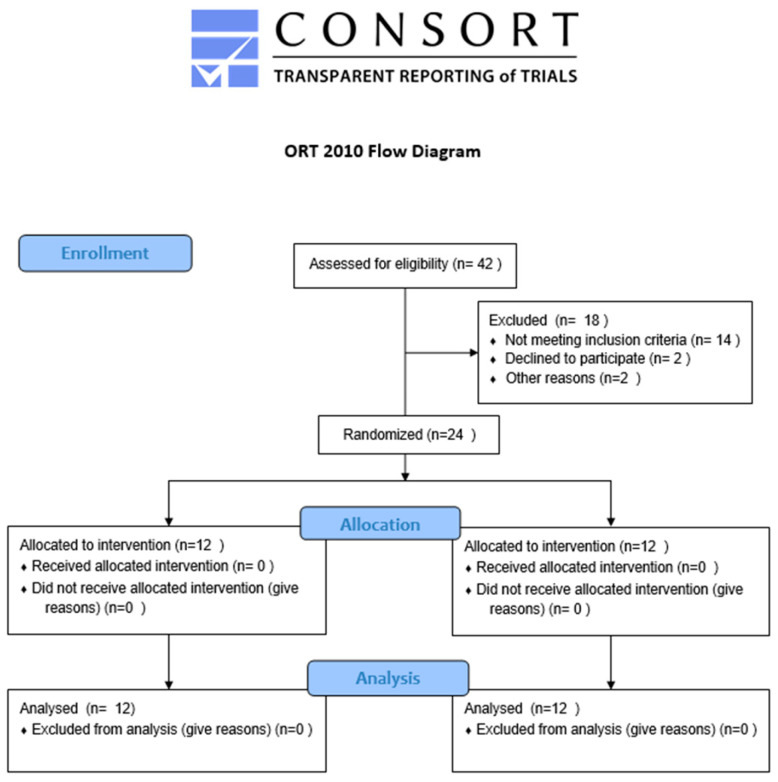
Flow diagram of participants through the stages of the study according to the CONSORT guidelines.

Due to its exploratory nature and strict inclusion criteria (age, sex, and health status), the study assessed a total of 30 individuals for eligibility. Six participants were excluded due to failure to meet the inclusion criteria or refusal to participate. The remaining 24 participants were randomized into two equal-sized groups: 12 received the functional training intervention, and 12 were the control group. The statistical sample size (n = 24) was chosen due to the availability of eligible participants within the specified recruitment timeframe (Figure 3).

## 3. Results

### 3.1. Intergroup Comparison of Variables Before and After 12 Weeks

Based on the statistical analysis of the results in Group 1 and Group 2 in the initial study, no statistically significant differences (*p* > 0.05) were observed between the groups across all analyzed variables. After the intervention, the groups differed significantly. Based on the statistical analysis of the effect size in Group 1 and Group 2 in the initial study, no significant effect sizes were observed between the groups across all analyzed variables. After the intervention, effect sizes indicated substantial differences between the groups, particularly for VAS, δ_RHnαLA180_, δ_LHnαLA180_, ζ_αLAcRH30_, ζ_αLAcLH30_, t_RH30αLA180_, and t_LH30αLA180_. These results demonstrate a clear increase in effect sizes post-intervention, highlighting meaningful differences in the intervention impact between Group 1 and Group 2 (Table 2).

### 3.2. Indicators Enabling Qualitative Analysis of Forward Trunk Bending—Intragroup Comparison Before and After 12 Weeks

The statistical analysis showed significant differences in all examined variables before and after the therapy in Group 1 participants. In Group 2, no statistically significant changes were observed (Table 3). Before the training in Group 1, the average pain level was 5.5, which decreased to an average of 1.67 after 12 weeks. There was a significant reduction in the pelvic anterior tilt angle (βP_ATn_) in the neutral position in participants undergoing motor control training (G1), from an average of 15.22° to 11.84°. Analyzing δ_RHnαLA180_ and δ_LHnαLA180_ in Group 1, an average range of motion approximately 12° greater was noted in the right and left hip joints before reaching the semi-full angle in the lumbar spine. Before therapy, the change in the lumbar angle relative to the neutral position at 30° hip flexion (ζα_LAcRH30_, ζα_LAcLH30_) averaged 32.06° for the right hip joint, decreasing to an average of 20.43° after therapy. The average for the left hip joint was 31.66° before therapy, decreasing to 20.47° afterwards. These results are absolute values. Analysis of t_RH30αLA180_ and t_RH30αLA180_ in Group 1 showed that before the intervention, on average, the lumbar angle reached 180° 0.12 s earlier relative to movement in the right hip joint and 0.19 s later after therapeutic intervention. For the left hip joint, the corresponding values were 0.11 s earlier before the intervention and 0.20 s later afterwards. Based on the effect size analysis in G1 and G2 before and after the intervention, significant changes were observed in Group 1 across most analyzed variables. The largest effect sizes in Group 1 were noted for VAS (d = 1.80), α_LAn_ (d = 1.08), ζ_αLAcRH30_ (d = 1.57), ζ_αLAcLH30_ (d = 1.56), t_RH30αLA180_ (d = 1.11), and t_LH30αLA180_. Negative effect sizes were observed for β_PATn_ (d = −1.40), δ_RHnαLA180_ (d = −0.82), and δ_LHnαLA180_ (d = −0.76). In Group 2, the effect sizes were generally smaller. These findings indicate larger effect sizes in Group 1 across most variables, reflecting more substantial changes within this group after the intervention compared to Group 2.

## 4. Discussion

The creation of proprietary indicators determining the angles between individual segments of the lumbopelvic–hip complex has enabled a specific analysis of forward trunk bending motion. This is important for planning therapy for individuals with chronic lumbar spine pain. The proper timing of the lumbopelvic–hip rhythm is crucial for maintaining correct biomechanics of movement. This reduces the load on individual anatomical structures, decreasing the risk of injury. In cases of discomfort caused by poor posture, it is possible to restore the most optimal “neutral zone” for movement biomechanics [15,16]. A threshold value of 30° was used to analyze the relationships between the lumbar spine and the hip joints. This is based on the forward trunk bending pattern. Within this range, a person should be able to perform the movement through hip flexion while maintaining a neutral lumbar spine position and avoiding excessive movement in these spinal segments [17]. The increase in the lumbar angle αLAn by approximately 5° observed in Group 1 indicates that the participants learned to control the lumbar segment, maintaining this angle in a neutral position during the initial phase of trunk flexion. Awareness and control of the αLAn angle during functional movements, which begins with assuming a neutral position (developed awareness at the neurophysiological level), translates into the quality of these movements.

In their meta-analysis, Chun et al. [18] noted a reduction in lumbar lordosis in patients with chronic lumbar spine pain. This aligns with the observations in our own study, where functional training led to an increase in lumbar lordosis in our participants, as assessed by the designated lumbar angle. In the control group, changes were minor and insignificant. The differences in lumbar angles between the group undergoing motor control training and the controls at the beginning of the study were moderate (d ≈ 0.33), which may result from the greater pain severity and limited motor control in G1 participants. A smaller lumbar angle in this group could be a defensive response of the body to pain, as confirmed by previous studies indicating a reduction in lumbar lordosis angle in individuals with chronic back pain. The VAS results (d = 0.57) also suggest greater pain intensity in G1, even though the difference was not statistically significant. Post-intervention, both groups experienced reduced pain; however, a significantly greater positive effect was observed in the experimental group. Li et al. [19] indicate the effectiveness of the training in reducing chronic low back pain. They also highlight a significant improvement in functional capacity and a reduction in the level of disability in the participants. These findings are consistent with our observations. In contrast, Trybulski et al. [20] conducted a meta-analysis on the effects of isolated lumbar extension exercises on pain levels and functional performance. Although they demonstrated a significant reduction in pain, no improvement in functional capacity was observed following this type of intervention. Our findings, however, indicate an enhancement in the execution of functional movement patterns as a result of the applied exercises, which may contribute not only to temporary pain relief, but, more importantly, to preventing degenerative spinal changes and associated future pain. The effect sizes observed in our study (d = 1.80 for pain reduction) further emphasize the clinical significance of the functional training, which goes beyond statistical significance and represents meaningful changes in patients’ condition.

Malarvizhi et al. [21] report that individuals suffering from chronic lumbar spine pain exhibit increased anterior pelvic tilt. They associate this with a lack of pelvic control and weak trunk muscle activity, which can contribute to pain. In this project involving women undergoing training, the engagement of trunk muscles influenced the shaping of the natural lumbar lordosis curvature while simultaneously reducing the increased anterior pelvic tilt, β_PATn_, which initially in G1 was approximately 15.22°, changing by about 4°. The angles δ_RHnαLA180_ and δ_LHnαLA180_ indicate how much movement occurred in the hip joints before the lumbar lordosis flattened. After the intervention in G1, an increase of about 12° in the range of motion in the hip joints during the initial phase of the forward trunk bending pattern was observed. Concurrently, there was a reduction in movement in the lumbar segment during this time.

Takahashi et al. [22] studied healthy individuals using the Polhemus 3 Space-Fastrak magnetic three-dimensional motion analysis system and a needle pressure sensor to measure intradiscal pressure. This enabled them to examine the impact of body posture and forward trunk bending on the generated loads on lumbar spine structures. They indicated that a 30° forward trunk bending motion caused an approximate 360% increase in pressure on the intervertebral disk compared to the neutral position. Their study identified one of the causes of pain, injury, or intervertebral disk herniation as improper and non-ergonomic execution of daily activities involving forward trunk bending. The authors’ observations are consistent with our findings. The designated indicators ζ_αLAcRH30_ and ζ_αLAcLH30_ determine the amount of movement in the lumbar spine during the initial phase (up to 30° of hip joint movement) of the forward trunk bending pattern. The introduction of functional training of the lumbopelvic–hip complex reduced lumbar spine movement by approximately 12° in the first 30° of the pattern. This directly translates into generating lower loads on the intervertebral disks. Our findings showed very large effect sizes (d = 1.57 for right side and d = 1.56 for left side) for the reduction in lumbar movement during the initial phase of forward bending, indicating a clinically significant improvement in movement pattern. No such changes were observed in the control group; on the contrary, an increase in the average value was noted, indicating that lack of rehabilitation may consequently worsen motor control compromised by chronic pain.

The proprietary indicators t_RH30αLA180_ and t_LH30αLA180_ used in our study allowed us to assess the time difference [s] between the moment when the right and left hip joint angles reach 30° relative to the neutral position and the moment when the lumbar angle reaches 180°. Group 1 results indicate the initiation and greater involvement of the hip joints in movement during forward trunk bending compared to the lumbar spine movement in the women undergoing therapy. This corresponds to a change in the forward trunk bending pattern and improved lumbopelvic–hip rhythm. In the control group (G2), there was a tendency for the lumbar angle to reach the full angle earlier, indicating a deterioration in movement technique analysis. These indicators are crucial from the perspective of treating and preventing chronic pain. Tsang et al. [23] clearly demonstrated that the kinematics of movement and the lumbopelvic–hip rhythm pattern are related to the speed of forward bending motion. The ability to control it was found only in individuals without pain, while those with chronic lumbar spine pain performed this pattern in a stereotypical, disrupted manner, for example, initiating the movement with lumbar spine flexion. This could be a key factor contributing to the persistence of pain. An absolute change of approximately 0.31 s in the onset of lumbar spine involvement during forward trunk bending in the women who underwent functional training indicates a significant improvement in motor control skills. The significant improvement observed in the experimental group in t_RH30αLA180_ (d = 2.21) and t_LH30αLA180_ (d = 2.18) after the intervention represents an extremely large effect, far exceeding the threshold of d = 0.8 typically considered large. Such substantial effect sizes highlight the profound clinical significance of the observed changes in movement coordination. These improvements likely resulted from targeted training, which improved coordination and timing of movement between the lumbar, pelvic, and hip segments. This improvement may have reduced spinal loads during trunk flexion, as supported by Tojima et al., who demonstrated that improper motion patterns increase the load on lumbar structures, leading to pain and strain [24].

Overall, the effect sizes observed across multiple parameters in this study (ranging from d = 0.76 to d = 2.37) highlight the robust efficacy of the functional training intervention, with effects that are not only statistically significant but also represent substantial clinical improvements. Changing the movement pattern, thereby reducing the load generated on the lumbar spine during the initial phase of movement, can be a significant factor in alleviating lumbar spine pain and preventing its exacerbation in the future.

### Study Limitations

This study has several limitations; future research would benefit from a larger study group, including men and different age groups. Additionally, the creation of additional indicators using the BTS SMART-D tool could be considered to precisely assess the impact of anterior and posterior pelvic tilt, as well as the position of the pelvis relative to the feet, on the biomechanics of movement in the participants.

## 5. Conclusions

The targeted motor control training proved effective in reducing pain and improving motor functions. A significant modification in the quality and technique and the angular relationships in the lumbopelvic–hip complex during the forward trunk bending pattern was observed in women who underwent functional training.

## Figures and Tables

**Table 1:** Characteristics of participants in Group 1 and Group 2.

	Group	$\bar{x}$ ± SD	Me	Min–Max	*p*
**Age [years]**	G1	21.92 ± 1.00	22.00	21.00–24.00	1.00
	G2	21.92 ± 1.00	22.00	21.00–24.00	
**Height [cm]**	G1	162.67 ± 6.28	162.50	150.00–170.00	0.65
	G2	163.75 ± 5.32	162.50	158.00–174.00	
**Body Mass [kg]**	G1	58.50 ± 8.11	55.50	42.00–69.00	0.22
	G2	62.00 ± 5.20	61.50	55.00–70.00	
**BMI [kg/m^2^]**	G1	22.03 ± 2.17	21.95	18.67–25.97	0.14
	G2	23.09 ± 0.94	23.03	21.48–24.51	

$\bar{x}$—arithmetic mean; Me—median; Min—minimum value; Max—maximum value; SD—standard deviation; *p* < 0.05—statistically significant differences; BMI—body mass index.

**Table 2:** Intergroup comparison of variables before and after 12 weeks.

G1 vs. G2		*p*	d	G1 vs. G2		*p*	d
Before	**VAS**	0.79	0.11	Before	**ζ_αLAcRH30_**	0.13	−0.64
After		0.00 *	−2.37	After		0.01 *	1.05
Before	**α_LAn_**	0.19	−0.55	Before	**ζ_αLAcLH30_**	0.11	−0.67
After		0.87	−0.07	After		0.03 *	0.98
Before	**β_PATn_**	0.19	0.55	Before	**t_RH30αLA180_**	0.70	−0.16
After		0.74	−0.14	After		0.00 *	2.21
Before	**δ_RHnαLA180_**	0.43	0.33	Before	**t_LH30αLA180_**	0.63	−0.20
After		0.01 *	−1.08	After		0.00 *	2.18
Before	**δ_LHnαLA180_**	0.39	0.35				
After		0.01 *	−1.10				

VAS—pain intensity; αLAn—lumbar angle [°]; βPATn—pelvic anterior tilt [°]; δRHnαLA180/δLHnαLA180 [°]—angle change in the right and left hip joint [°] between the neutral position and the one achieved at a lumbar angle of 180°; ζαLAcRH30/ζαLAcLH30—lumbar angle change [°] relative to the neutral position at 30° of flexion in the right and left hip joint; tRH30αLA180/tLH30αLA180—time difference [s] between the point where the angle change relative to the neutral position in the right and left hip joint reaches 30° and the point where the lumbar angle reaches 180°; * *p* < 0.05—statistically significant differences; d—effect size.

**Table 3:** Values of indicators enabling qualitative analysis of forward trunk bending movement. Intragroup comparison before and after 12 weeks.

		Group	$\bar{x}$ ± SD	Me	Min–Max	*p*	d
**VAS**	Before	G1	5.50 ± 1.73	5.50	3.00–8.00	0.00 *	1.80
	After		1.67 ± 0.89	1.67	0.00–3.00		
	Before	G2	5.33 ± 1.23	5.00	4.00–7.00	0.09	0.57
	After		4.42 ± 1.38	4.00	3.00–7.00		
**α_LAn_** [°]	Before	G1	150.66 ± 7.69	150.41	138.27–161.16	0.00 *	1.08
	After		155.97 ± 8.18	157.09	142.14–168.20		
	Before	G2	154.53 ± 6.21	157.39	141.51–160.57	0.27	0.33
	After		156.56 ± 8.78	158.10	140.54–169.98		
**β_PATn_** [°]	Before	G1	15.22 ± 4.20	14.63	9.29–23.74	0.00 *	−1.40
	After		11.84 ± 3.75	11.57	5.58–17.27		
	Before	G2	12.78 ± 4.68	12.21	7.36–23.74	0.86	−0.05
	After		12.46 ± 6.32	11.83	5.52–23.78		
**δ_RHnαLA180_** [°]	Before	G1	24.63 ± 10.37	23.13	4.89–39.84	0.02 *	−0.82
	After		36.69 ± 14.31	34.99	12.73–56.71		
	Before	G2	27.88 ± 9.63	39.88	14.37–39.88	0.22	0.37
	After		24.33 ± 7.57	39.11	8.44–39.11		
**δ_LHnαLA180_** [°]	Before	G1	25.63 ± 10.49	24.05	5.86–41.67	0.02 *	−0.76
	After		37.41 ± 15.18	36.26	13.77–60.05		
	Before	G2	29.07 ± 8.82	29.75	17.32–41.67	0.08	0.54
	After		24.27 ± 7.28	24.68	8.25–37.32		
**ζ_αLAcRH30_** [°]	Before	G1	32.06 ± 5.86	30.36	24.18–42.51	0.00 *	1.57
	After		20.43 ± 6.32	21.62	11.36–29.82		
	Before	G2	27.84 ± 7.31	28.47	16.87–38.68	0.63	−0.14
	After		29.31 ± 10.21	29.21	11.37–42.51		
**ζ_αLAcLH30_** [°]	Before	G1	31.66 ± 6.24	31.08	21.56–44.19	0.00 *	1.56
	After		20.47 ± 6.76	21.19	10.74–29.82		
	Before	G2	27.25 ± 6.89	28.79	16.87–38.68	0.54	−0.18
	After		29.22 ± 10.69	30.09	10.58–44.19		
**t_RH30αLA180_** [s]	Before	G1	−0.12 ± 0.21	−0.10	−0.60–0.19	0.00 *	1.11
	After		0.19 ± 0.21	0.15	−0.08–0.50		
	Before	G2	−0.09 ± 0.23	−0.04	−0.56–0.16	0.08	−0.57
	After		−0.13 ± 0.18	−0.20	−0.69–−0.03		
**t_LH30αLA180_** [s]	Before	G1	−0.11 ± 0.22	−0.12	−0.64–0.17	0.00 *	1.12
	After		0.20 ± 0.21	0.15	−0.07–0.50		
	Before	G2	−0.07 ± 0.20	−0.02	−0.53–0.16	0.06	−0.63
	After		−0.12 ± 0.18	−0.18	−0.64–−0.01		

αLAn—lumbar angle [°]; βPATn—pelvic anterior tilt [°]; δRHnαLA180/δLHnαLA180 [°]—angle change in the right and left hip joint [°] between the neutral position and the one achieved at a lumbar angle of 180°; ζαLAcRH30/ζαLAcLH30—lumbar angle change [°] relative to the neutral position at 30° of flexion in the right and left hip joint; tRH30αLA180/tLH30αLA180—time difference [s] between the point where the change in the angle value relative to the neutral position in the right and left hip joint reaches 30° and the point where the lumbar angle reaches 180°; VAS—pain intensity; * *p* < 0.05—statistically significant difference; d—effect size.

## Data Availability

Data are available on request from the corresponding author.

## Supplementary Materials

The following supporting information is available at https://www.mdpi.com/article/10.3390/jcm14124156/s1, Table S1. A general strategy for functional training of the lumbopelvic–hip complex intended for Group 1.

## References

[1] Emorinken A., Erameh C.O., Akpasubi B.O., Dic-Ijiewere M.O., Ugheoke A.J. (2023). Epidemiology of low back pain: Frequency, risk factors, and patterns in South-South Nigeria. Reumatologia.

[2] Claeys K., Brumagne S., Dankaerts W., Kiers H., Janssens L. (2011). Decreased variability in postural control strategies in young people with non-specific low back pain is associated with altered proprioceptive reweighting. Eur. J. Appl. Physiol..

[3] Schmid S., Bangerter C., Schweinhardt P., Meier M.L. (2021). Identifying Motor Control Strategies and Their Role in Low Back Pain: A Cross-Disciplinary Approach Bridging Neurosciences with Movement Biomechanics. Front. Pain Res..

[4] Schouppe S., Clauwaert A., Van Oosterwijck J., Van Damme S., Palmans T., Wiersema J.R., Sanchis-Sanchéz E., Danneels L. (2020). Does experimentally induced pain-related fear influence central and peripheral movement preparation in healthy people and patients with low back pain?. Pain.

[5] Feng L., Li H., Cui H., Xie X., Xu S., Hu Y. (2021). Low Back Pain Assessment Based on Alpha Oscillation Changes in Spontaneous Electroencephalogram (EEG). Neural Plast..

[6] Teixeira P.E.P., Pacheco-Barrios K., Uygur-Kucukseymen E., Machado R.M., Balbuena-Pareja A., Giannoni-Luza S., Luna-Cuadros M.A., Cardenas-Rojas A., Gonzalez-Mego P., Mejia-Pandoet P.F. (2022). Electroencephalography Signatures for Conditioned Pain Modulation and Pain Perception in Nonspecific Chronic Low Back Pain-An Exploratory Study. Pain Med..

[7] Gervasio S., Zarei A.A., Mrachacz-Kersting N. (2023). EEG signatures of low back and knee joint pain during movement execution: A short report. Front. Rehabil. Sci..

[8] Eto K., Wake H., Watanabe M., Ishibashi H., Noda M., Yanagawa Y., Nabekura J. (2011). Inter-regional contribution of enhanced activity of the primary somatosensory cortex to the anterior cingulate cortex accelerates chronic pain behavior. J. Neurosci..

[9] Maharty D.C., Hines S.C., Brown R.B. (2024). Chronic Low Back Pain in Adults: Evaluation and Management. Am. Fam. Physician.

[10] Wasik J. (2009). Structure of movement of a turning technique used in the event of special techniques in Taekwon-do ITF. Arch. Budo.

[11] Byrom B., Elash C.A., Eremenco S., Bodart S., Muehlhausen W., Platko J.V., Watson C., Howry C. (2022). Measurement Comparability of Electronic and Paper Administration of Visual Analogue Scales: A Review of Published Studies. Ther. Innov. Regul. Sci..

[12] Czamara A., Markowska I., Hagner-Derengowska M. (2015). Three-dimensional kinematic analysis of ankle, knee, hip, and pelvic rotation during gait in patients after anterior cruciate ligament reconstruction—Early results. BMC Musculoskelet. Disord..

[13] Jochymczyk-Woźniak K., Nowakowska-Lipiec K., Zadoń H., Wolny S., Gzik M., Gorwa J., Michnik R. (2020). Gait Kinematics Index, Global Symmetry Index and Gait Deviations Profile: Concept of a new comprehensive method of gait pathology evaluation. Acta Bioeng. Biomech..

[14] Sullivan G.M., Feinn R. (2012). Using Effect Size-or Why the P Value Is Not Enough. J. Grad. Med. Educ..

[15] Sahrmann S.A. (2002). Diagnosis and Treatment of Movement Impairments Syndromes.

[16] Panjabi M.M. (1992). The stabilizing system of the spine. I: Function, dysfunction, adaptation, and enhancement. J. Spinal Disord..

[17] Comerford M., Mottram S. (2012). Kinetic Control. The Management of Uncontrolled Movement.

[18] Chun S.W., Lim C.Y., Kim K., Hwang J., Chung S.G. (2017). The relationships between low back pain and lumbar lordosis: A systematic review and meta-analysis. Spine J..

[19] Li Y., Zhao Q., Zhang X.E.Y., Su Y. (2025). The impact of core training combined with breathing exercises on individuals with chronic non-specific low back pain. Front. Public Health.

[20] Trybulski R., Michał W., Małgorzata S., Bogdański B., Bichowska-Pawęska M., Ryszkiel I., Gepfert M., Clemente F.M. (2025). Impact of isolated lumbar extension strength training on reducing nonspecific low back pain, disability, and improving function: A systematic review and meta-analysis. Sci. Rep..

[21] Malarvizhi D., Sai Kishore Varma R., Sivakumar V.P.R. (2017). Measurement of anterior pelvic tilt in low back pain—An observational study. Asian J. Pharm. Clin. Res..

[22] Takahashi I., Kikuchi S., Sato K., Sato N. (2006). Mechanical load of the lumbar spine during forward bending motion of the trunk-a biomechanical study. Spine.

[23] Tsang S.M.H., Szeto G.P.Y., Li L.M.K., Wong D.C.M., Yip M.M.P., Lee R.Y.W. (2017). The effects of bending speed on the lumbo-pelvic kinematics and movement pattern during forward bending in people with and without low back pain. BMC Musculoskelet. Disord..

[24] Tojima M., Ogata N., Inokuchi H., Haga N. (2016). Three-dimensional motion analysis of lumbopelvic rhythm during lateral trunk bending. J. Phys. Ther. Sci..

