# Supplementary material for: Analysis of Forward Trunk Bending in Women with Chronic Low Back Pain Undergoing Functional Training
Source: J Clin Med. 2025 Jun 11;14(12):4156. doi: 10.3390/jcm14124156 (PMC12194704; doi:10.3390/jcm14124156)
Supplement: Supplementary file 1 [file jcm-14-04156-s001.zip › jcm-3468974-supplementary.pdf]

	12-WEEK EXERCISE PROGRAM												
**1. MOTOR CONTROL AND STABILITY**	**Explanation**	**Week 1**	**Week 2**	**Week 3**	**Week 4**	**Week 5**	**Week 6**	**Week 7**	**Week 8**	**Week 9**	**Week 10**	**Week 11**	**Week 12**
1.1 Neutral Pelvic Position	Practice maintaining a neutral pelvic position while standing or lying down. This exercise focuses on developing awareness and control of the pelvis's alignment relative to the lumbar spine.	2x10	3x10	-	-	-	-	-	-	-	-	-	-
1.2 Trunk Tilt	Perform a controlled trunk tilt forward and backward while keeping the pelvis stable. This movement improves the coordination between the lumbar spine and the pelvis.	2x15	3x15	4x15	4x15	-	-	-	-	-	-	-	-
1.3 Small Knee Band	Perform exercises with a small knee band to activate and strengthen the hip abductors and stabilizers. Focus on maintaining proper alignment of the pelvis and spine during movement.	2x15	3x15	2x15	2x15	-	-	-	-	-	-	-	-
1.3a Small Knee Band (Single Leg)	Execute single-leg movements with the knee band to increase the load on one side, promoting unilateral stability and pelvic control.	-	-	2x15x15	2x15x15	3x15x15	3x15x15	4x15x15	4x15x15	-	-	-	-
1.4 Supine with Bent Legs (Leg Extension)	Begin in a supine position with knees bent. Extend one or both legs while maintaining a stable pelvis and lumbar spine to improve core and pelvic control.	-	-	2x10x10	2x12x10	3x10x10	3x12x12	3x14x14	4x10x10	4x12x12	4x14x14	-	-
1.5 Knee Drop while Supine with Pelvic	In a supine position, allow one knee to drop to the side	-	-	2x10x10	2x12x10	3x10x10	3x12x12	3x14x14	4x10x10	4x12x12	4x14x14	-	-

Control	while ensuring the pelvis remains stable. This exercise enhances pelvic stability during asymmetrical movements.												
1.6 Quadruped Position	Assume a quadruped (all fours) position, maintaining a neutral spine and stable pelvis. This is the base position for subsequent progressions.	2x10	3x10	-	-	-	-	-	-	-	-	-	-
1.6a Quadruped Position + Upper Limb Extension	Extend one upper limb while keeping the pelvis and spine stable. This exercise improves upper-limb coordination and core stability.	-	-	3x10x10	3x12x12	4x10x10	4x12x12	-	-	-	-	-	-
1.6b Quadruped Position + Lower Limb Extension	Extend one lower limb, focusing on maintaining pelvic alignment and preventing lumbar hyperextension. This targets lower-limb coordination and core control.	-	-	-	-	-	-	3x12x12	4x10x10	4x12x12	-	-	-
1.6c Quadruped Position + Opposite Upper and Lower Limb Extension	Extend the opposite upper and lower limbs simultaneously, maintaining a stable core and pelvis. This advanced movement promotes cross-body coordination and core stability.	-	-	-	-	-	-	-	-	-	3x10x10	4x10x10	4x12x12
1.7 Hip Flexion in Sitting with a Bent Knee	In a seated position, flex the hip with the knee bent, ensuring the pelvis remains neutral. This exercise targets the hip flexors and pelvic control in a functional position.	2x10x10	2x12x12	2x14x14	3x10x10	3x12x12	3x14x14	4x10x10	4x12x12	4x14x14	4x16x16	4x16x16	4x16x16
1.8 Controlled "Sitting	From a quadruped	-	-	-	-	-	-	-	-	3x10x10	3x12x12	4x10x10	4x12x12

on Heels" in Quadruped	position, move into a controlled "sitting on heels" movement, focusing on maintaining pelvic alignment and lumbar stability throughout the motion.												
**2. STRENGTHENING**	**Explanation**	**Week 1**	**Week 2**	**Week 3**	**Week 4**	**Week 5**	**Week 6**	**Week 7**	**Week 8**	**Week 9**	**Week 10**	**Week 11**	**Week 12**
2.1 Bridge	Perform a bridge by lifting the pelvis from a supine position off the ground, activating the gluteal and core muscles.	3x12	3x12	3x12	3x12	2x10	2x8	1x12	1x10	1x8	1x8	1x8	1x8
2.1a Bridge + Leg Extension	While maintaining the bridge position, extend one leg at the knee without losing pelvic alignment. This progression enhances unilateral stability and strength.	-	-	2x10x10	2x10x10	3x10x10	3x10x10	3x12x12	3x12x12	3x14x14	4x10x10	4x12x12	4x14x14
2.2 Squat	Execute a standard squat, focusing on properly aligning the knees, hips, and spine to engage the quadriceps, glutes, and core.	-	-	2x15x15	2x15x15	3x15x15	3x15x15	4x15x15	4x15x15	-	-	-	-
2.2a Squat with Band Overhead	Perform a squat while holding a resistance band overhead to activate the upper body and improve core engagement and postural stability.	-	-	-	-	-	-	-	-	2x15x15	3x15x15	3x15x15	4x15x15
2.3 Deadlift	Perform a traditional deadlift, maintaining a neutral spine and engaging the posterior chain muscles, including the hamstrings, glutes, and lower back.	-	-	-	-	4x15	4x15	5x15	5x15	-	-	-	-
2.3a Deadlift with Thera Band	Perform a modified deadlift using a Thera Band to	-	-	-	-	-	-	-	-	4x15	4x15	5x15	5x15

	provide resistance, enhancing control and activation of the posterior chain.												
2.4 External Hip Rotation in Side-Lying	Lie on one side and perform external rotation of the top leg at the hip joint. This exercise targets the external rotators and improves hip stability.	2x10x10	2x12x12	2x14x14	3x10x10	3x12x12	3x14x14	4x10x10	4x12x12	4x14x14	4x16x16	4x16x16	4x16x16
2.5 Hip Extension with Bent Knee in Prone	Lie prone and extend the hip while keeping the knee bent, engaging the gluteus maximus and hamstrings.	-	-	3x12	3x12	3x14x14	3x14x14	4x10x10	4x10x10	4x12x12	4x12x12	4x14x14	4x14x14
2.6 Dead Bug	Lie on your back with arms and legs extended toward the ceiling. Move opposite arms and legs toward the floor while maintaining core stability.	4x30s	4x30s	2x30s	2x30s	2x35s	2x35s	1x30s	1x30s	1x30s	1x30s	1x30s	1x30s
2.6a Dead Bug + Lowering Both Legs	Perform the dead bug while lowering both legs simultaneously, ensuring the pelvis remains stable, and the lower back does not arch.	-	-	2x15	2x15	1x15	1x15	1x10	1x10	-	-	-	-
2.6b Dead Bug + Lowering One Leg	Perform the dead bug while lowering one leg at a time, maintaining core engagement and pelvic stability.	-	-	-	-	2x15x15	2x15x15	3x15x15	3x15x15	4x10x10	4x10x10	4x15x15	4x15x15
2.6c Dead Bug + Isometric Hold	Hold the arms and legs in an extended position while maintaining core activation, emphasizing isometric control.	-	-	-	-	3x5sx5s	3x5sx5s	3x10sx10s	3x10sx10s	3x10sx10s	4x8sx8s	4x10sx10s	4x10sx10s
2.7 Pallof Press with Extension	Perform a Pallof press while extending the arms fully, resisting rotation, and engaging the core and oblique muscles.	-	-	-	-	-	-	-	-	3x12x12	3x12x12	4x10x10	4x10x10

3. STRETCHING	Explanation	Week 1	Week 2	Week 3	Week 4	Week 5	Week 6	Week 7	Week 8	Week 9	Week 10	Week 11	Week 12
3.1 Hamstring Muscles	Perform a static or dynamic stretch for the hamstring muscles. Sit with one leg extended and the other bent, reaching toward the toes of the extended leg while maintaining a straight back. This exercise increases flexibility in the posterior thigh.	4x5sx5s	4x5sx5s	5x5sx5s	5x5sx5s	4x8sx8s	4x8sx8s	5x8sx8s	5x8sx8s	4x10sx10s	4x10sx10s	5x10sx10s	5x10sx10s
3.2 Quadriceps Muscles	Stretch the quadriceps by pulling one ankle toward the glutes in a standing or side-lying position. Keep the pelvis neutral and avoid arching the lower back.	4x5sx5s	4x5sx5s	5x5sx5s	5x5sx5s	4x8sx8s	4x8sx8s	5x8sx8s	5x8sx8s	4x10sx10s	4x10sx10s	5x10sx10s	5x10sx10s
3.3 "Japanese Bow"	Assume a kneeling position and slowly lean forward, extending the arms out in front while keeping the hips close to the heels. This stretch targets the lumbar spine, lower back, and hip extensors.	-	-	-	-	-	-	3x14	3x14	4x12	4x12	5x12	5x12
3.4 Pectoral Muscles	Perform a doorway stretch by placing the forearms on the sides of a doorway and gently leaning forward. This opens the chest and stretches the pectoral muscles, promoting better posture and reducing tightness.	-	-	-	-	-	-	-	-	30 s	30 s	35 s	35 s
